# Transvesicoscopic Politano–Leadbetter Versus Laparoscopic Lich–Gregoir Ureteral Reimplantation for Primary Obstructive Megaureter in Infants and Toddlers: A Retrospective Comparative Cohort Study

**DOI:** 10.3390/jcm15166178

**Published:** 2026-08-10

**Authors:** Huazhang Liu, Minghui Pan, Liming Jin, Guangjie Chen, Chang Tao, Xiang Yan

**Affiliations:** Department of Urology, Children’s Hospital, National Clinical Research Center for Children and Adolescents’ Health and Diseases, Zhejiang University School of Medicine, No. 3333 of Binsheng Road, Hangzhou 310003, China; 6516146@zju.edu.cn (H.L.);

**Keywords:** primary obstructive megaureter, infants, toddlers, transvesicoscopic surgery, Politano–Leadbetter, Lich–Gregoir, ureteral reimplantation, minimally invasive surgery

## Abstract

**Objective:** Our objective was to compare clinical safety and postoperative efficacy between transvesicoscopic Politano–Leadbetter (TPL) and laparoscopic Lich–Gregoir (LLG) ureteral reimplantation in infants and toddlers under 36 months diagnosed with primary obstructive megaureter (POM). **Methods:** This was a single-center retrospective cohort study of pediatric patients with POM who underwent ureteral reimplantation between January 2018 and April 2025. Patients were stratified into TPL and LLG groups by surgical approach. Baseline characteristics, perioperative outcomes, perioperative complications, imaging findings, renal functional outcomes, and long-term complications were collected and analyzed. Primary group comparisons were unadjusted, with additional multivariable linear regression performed for operative duration. **Results:** We enrolled 65 children: 30 in the TPL group and 35 in the LLG group. Baseline characteristics showed no statistically significant differences across the two study cohorts. There were no open conversions in either group. Total operative duration did not differ significantly between the TPL and LLG groups (145.8 ± 30.6 min vs. 136.9 ± 25.2 min; mean difference, 8.9 min; 95% CI, −4.9 to 22.7; *p* = 0.210). In multivariable linear regression, TPL showed a non-significant trend toward longer operative time after adjustment for age, preoperative ureteral diameter, and surgery year (B = 10.4 min; 95% CI, −3.7 to 24.4; *p* = 0.141). Time to postoperative oral intake was slightly shorter in the TPL group (6.1 ± 2.3 h vs. 8.2 ± 2.9 h; mean difference, −2.1 h; 95% CI, −3.4 to −0.8; *p* = 0.002). No statistically significant differences were detected in estimated blood loss, ureteral tapering rate, double-J stent placement rate, postoperative pain score, acetaminophen use, urinary catheterization duration, or length of hospital stay. Perioperative complications occurred in 3 patients in the TPL group and 5 patients in the LLG group (10.0% vs. 14.3%; risk difference, −4.3%; 95% CI, −20.1% to 11.5%; *p* = 0.716). No Clavien–Dindo grade III or higher complications occurred in either group. During follow-up, both groups showed reductions in anteroposterior pelvic diameter (APD) and ureteral diameter (UD), with preserved differential renal function (DRF). Low-grade postoperative vesicoureteral reflux (VUR) was detected in two LLG patients, including one grade I case at 4 months and one grade II case at 6 months after surgery; both were managed conservatively. Neither group experienced recurrent ureterovesical junction obstruction (UVJO) nor required reoperation during follow-up. **Conclusions:** In this single-center retrospective cohort of infants and toddlers with POM, no statistically significant differences were detected in the main perioperative and follow-up outcomes between TPL and LLG. TPL enables intravesical reconstruction and better preserves the natural anatomical course of the ureter and may serve as an alternative minimally invasive option for this patient population.

## 1. Introduction

Primary obstructive megaureter (POM) is defined as ureteral dilatation caused by either functional or structural stenosis located at the distal ureterovesical junction. Most children with POM show spontaneous resolution or remain clinically stable during postnatal follow-up; therefore, initial conservative observation remains an important management strategy [1,2]. However, surgical intervention is still required in children with recurrent febrile urinary tract infections (UTI), progressive hydroureteronephrosis, or deterioration of differential renal function (DRF) [1,3].

Ureteral reimplantation is a classic surgical treatment for POM. This procedure relieves distal obstruction, improves upper urinary tract dilatation, and preserves ipsilateral renal function by excising the distal narrowed or adynamic ureteral segment, performing ureteral tapering when necessary, and reconstructing an antireflux submucosal tunnel of adequate length. Open ureteral reimplantation remains a well-established definitive treatment, particularly for markedly dilated ureters or complex anatomy [4]. With the development of pediatric minimally invasive surgery, laparoscopic extravesical reimplantation and transvesicoscopic intravesical reimplantation have increasingly been applied, with favorable perioperative outcomes reported in selected patients [5]. In infants requiring early intervention, temporary drainage with double-J stenting or nephrostomy may be used as a bridging strategy [6], while endoscopic balloon dilatation has also been reported as a less invasive alternative [7].

Among minimally invasive techniques, laparoscopic Lich–Gregoir reimplantation (LLG) is performed through an extravesical approach, is less dependent on bladder capacity, and provides a relatively wider intra-abdominal working space; therefore, LLG has been more commonly used for POM in young children. In contrast, transvesicoscopic Politano–Leadbetter reimplantation (TPL) allows ureteral mobilization, creation of a submucosal tunnel, and ureteral reimplantation to be completed intravesically. TPL enables reconstruction along a course that more closely approximates the natural anatomical trajectory of the ureter and may preserve the feasibility of future retrograde ureteral access. However, in young children, TPL remains technically demanding because of limited bladder capacity, restricted intravesical working space, and the need to maintain stable pneumovesicum [8,9].

Despite increasing interest in minimally invasive ureteral reimplantation, evidence regarding TPL for POM in infants and toddlers remains limited. Moreover, direct comparative data between TPL and LLG ureteral reimplantation in this specific population are scarce. Therefore, the present study aimed to compare the perioperative and follow-up outcomes of TPL and LLG for POM in infants and toddlers.

## 2. Materials and Methods

**Figure 1:**
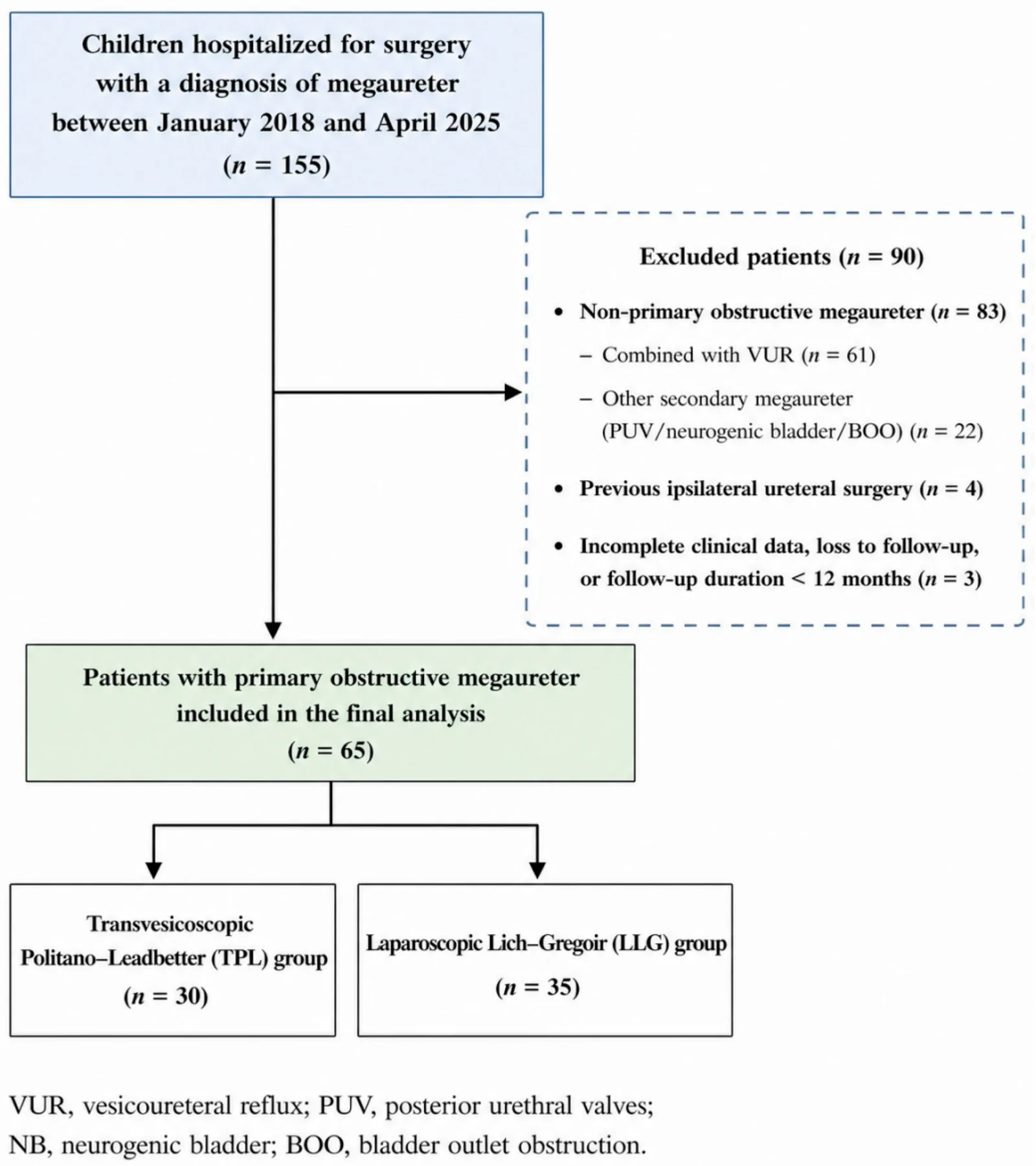
Flowchart of patient selection.

Consecutive infants and toddlers aged ≤36 months who were hospitalized for surgical treatment of POM at our center between January 2018 and April 2025 were retrospectively screened. The flowchart in Figure 1 details how participants were recruited. Of 155 initially screened patients, 65 with POM were included in the final analysis, including 30 who underwent TPL ureteral reimplantation and 35 who underwent LLG ureteral reimplantation.

Preoperative evaluation included magnetic resonance urography (MRU), urinary ultrasonography, voiding cystourethrography (VCUG), and technetium-99 m dimercaptosuccinic acid renal scintigraphy (DMSA). POM was diagnosed based on ureteral dilatation without vesicoureteral reflux (VUR) or identifiable secondary causes of ureteral obstruction or dilatation.

Inclusion criteria were recurrent febrile UTI, progressive renal pelvic and ureteral dilatation during follow-up, or deterioration in DRF. Exclusion criteria were non-primary obstructive megaureter, previous ipsilateral ureteral surgery, incomplete clinical data, loss to follow-up, or follow-up duration of less than 12 months.

Recurrent febrile UTI was defined as repeated febrile episodes (>38 °C) with pyuria and positive urine culture requiring antibiotic treatment. Progressive hydroureteronephrosis was defined as progressive persistent worsening of renal pelvic or ureteral dilatation on serial ultrasonography. Deterioration in DRF was defined as DRF <40% or a decrease of >10 percentage points between two examinations.

All procedures were performed by the same senior pediatric urologist experienced in minimally invasive ureteral reconstruction. During the study period, both procedures were routinely performed at our center. Before surgery, both TPL and LLG were explained to the parents as available minimally invasive surgical options, including the surgical route, potential advantages, limitations, and possible complications of each approach. No predefined preference for either approach was applied by the surgeons, and no consistent parental preference for one approach over the other was identified during the study period. The final surgical approach was determined through discussion between the surgeons and parents.

This study was approved by the Medical Ethics Committee of the Children’s Hospital, Zhejiang University School of Medicine (approval No. 2026-IRB-0207-P-01; approval date: 25 May 2026). The approval covered this retrospective cohort study comparing pneumovesical Politano–Leadbetter ureteral reimplantation and laparoscopic Lich–Gregoir ureteral reimplantation for primary obstructive megaureter in infants and young children. The requirement for informed consent was waived by the Medical Ethics Committee because this study used previously collected clinical data, involved no more than minimal risk, and included measures to protect patient privacy and personal information. Retrospective data extraction and analysis were performed after ethics approval had been obtained.

### 2.1. Surgical Techniques

#### 2.1.1. Transvesicoscopic Politano–Leadbetter Procedure

The patient was placed in the supine position under general anesthesia, with both lower limbs abducted approximately 45°. Cystoscopy was then performed, and CO_2_ was insufflated into the bladder to distend it to the level of the umbilicus while maintaining an intravesical pressure of 6–8 mmHg. The anterior bladder wall was suspended and fixed to the abdominal wall using 2–0 non-absorbable sutures. A 5 mm incision was made 1–2 cm caudal to the umbilicus for placement of a 30° laparoscope. Two additional 3 mm working ports were then placed on either side of the bladder.

Intraoperatively, both ureteral orifices were first inspected, and a 3F ureteral catheter was inserted into the affected ureter as a marker. After placement of a 5–0 non-absorbable traction suture at the distal end of the ureter, the ureter was dissected between the ureteral adventitia and the detrusor muscle, with careful release of the surrounding fibrotic tissue, until it could be mobilized into the bladder lumen without tension. The mobilized ureteral length was usually 3–5 cm, and particular attention was paid to preserving the ureteral blood supply throughout the dissection. The diseased distal ureteral segment was excised, and ureteral tapering was performed as appropriate according to the degree of ureteral dilatation.

**Figure 2:**
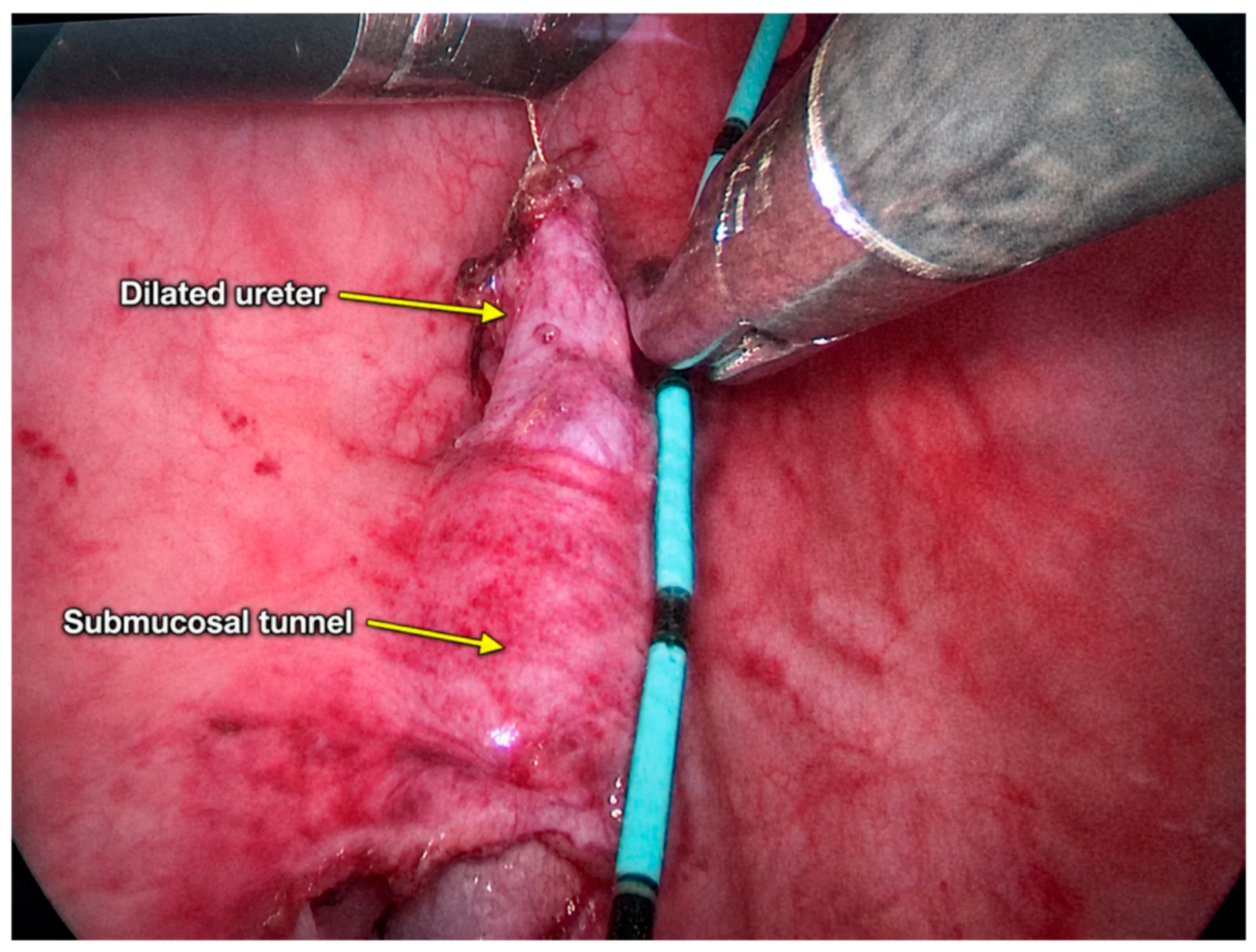
The ureter was pulled through the created submucosal tunnel during transvesicoscopic Politano–Leadbetter ureteral reimplantation.

A new ureteral hiatus was created proximal to the native ureteral orifice. The ureter was further mobilized and introduced into the bladder through the new hiatus, with careful protection of the surrounding structures, particularly the vas deferens. After ensuring that the ureter was free of tension or torsion, a submucosal tunnel was constructed between the new hiatus and the native ureteral orifice. The ureter was then passed through the tunnel and brought out through the native ureteral orifice (Figure 2).

The ureteral seromuscular layer was anchored to the adjacent bladder mucosa, with slight eversion of the ureteral mucosa to create a nipple-like ureteral orifice. Finally, at the new ureteral hiatus, the ureter was fixed to the detrusor muscle with interrupted sutures, and the bladder mucosal incision was closed.

#### 2.1.2. Laparoscopic Lich–Gregoir Procedure

The patient was placed supine under general anesthesia. A curved infraumbilical incision was made, and pneumoperitoneum was established (6–8 mmHg). A 5 mm camera port was established, and two 3 mm working trocars were introduced bilaterally beside the umbilicus under direct laparoscopic visualization.

The posterior peritoneum was cut over the iliac vasculature, followed by distal ureteral dissection down to the ureterovesical junction. The bladder was distended with 0.9% saline via the urethral catheter to maintain moderate distension and was suspended using 3–0 absorbable sutures. A longitudinal detrusorotomy was made at the ureterovesical junction, followed by careful separation of the detrusor from the bladder mucosa to form an extravesical submucosal tunnel. The submucosal tunnel was constructed to be approximately three to five times the ureteral diameter in length.

**Figure 3:**
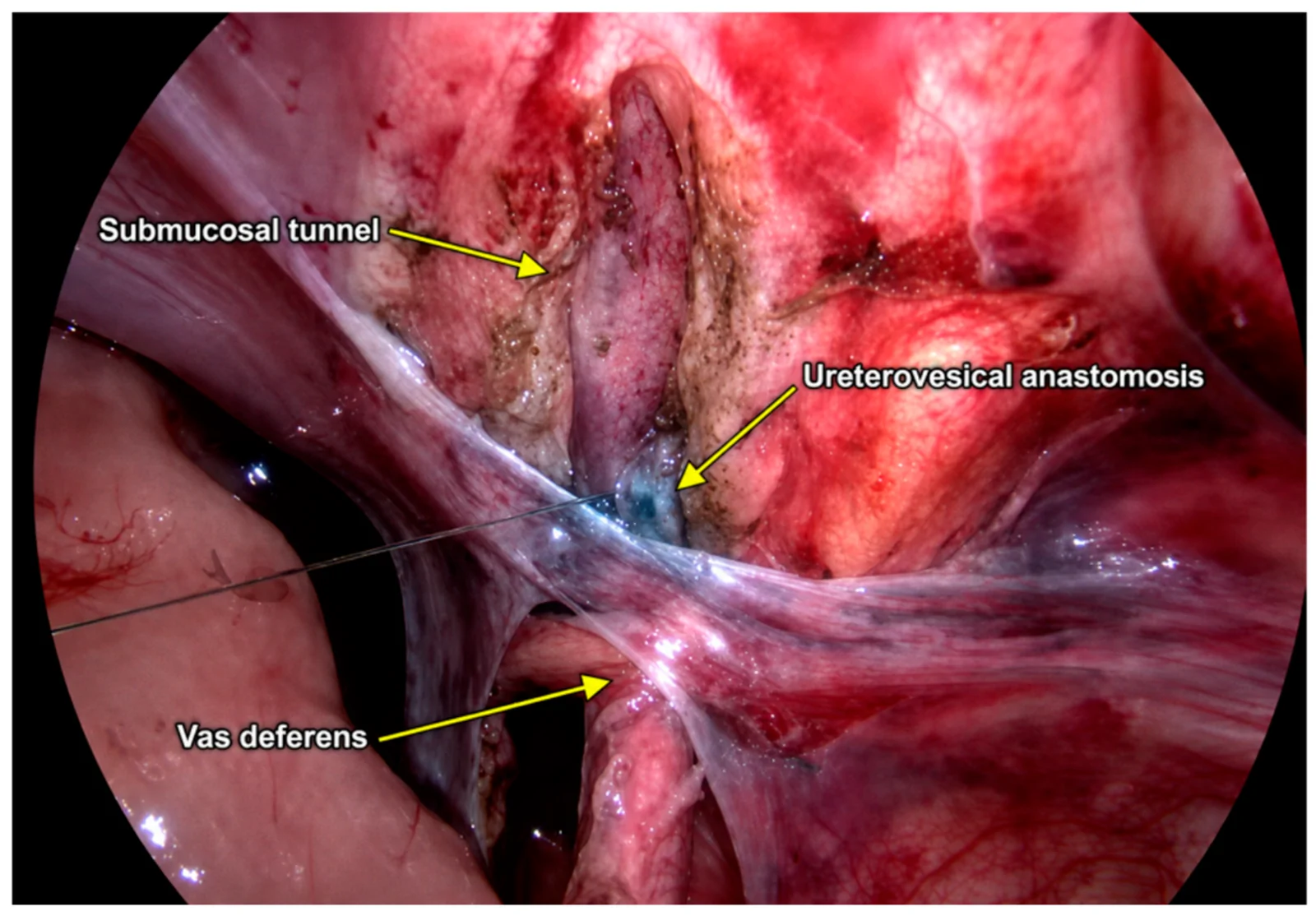
The extravesical submucosal tunnel was created by detrusor incision, and ureterovesical anastomosis was performed.

After distal transection at the ureterovesical junction, the abnormal terminal ureter was removed. Ureteral tapering was performed when indicated, depending on the severity of ureteral dilation. A new ureteral orifice was fashioned at the distal end of the tunnel, after which ureteroneocystostomy was completed with 6–0 sutures (Figure 3). The detrusor was finally reapproximated over the ureter using a continuous 4–0 barbed suture, thereby embedding the ureter within the bladder wall.

#### 2.1.3. Outcome Measures and Follow-Up

Preoperative baseline characteristics, perioperative variables, postoperative complications, and follow-up outcomes were collected and compared between the two groups. Preoperative baseline characteristics included age at surgery, body weight, sex, laterality, prenatal detection, postnatal symptomatic diagnosis, and preoperative temporary drainage.

Perioperative variables included total operative duration, estimated blood loss, ureteral tapering, time to postoperative oral intake, Face, Legs, Activity, Cry, and Consolability (FLACC) pain score on postoperative day 1, postoperative acetaminophen use, duration of urinary catheterization, length of hospital stay, and perioperative adverse events. In the TPL group, total operative duration included cystoscopic guidance, establishment of pneumovesicum, trocar placement, intravesical ureteral mobilization, ureteral tapering when required, ureteral reimplantation, and wound closure. In the LLG group, total operative duration included establishment of pneumoperitoneum, trocar placement, ureteral mobilization, ureteral tapering when required, extravesical ureteral reimplantation, and wound closure.

Postoperative pain was assessed using the FLACC pain scale, and the score on postoperative day 1 was recorded. Acetaminophen was administered as needed according to our institutional analgesic protocol, mainly when the FLACC score was ≥4 or when caregivers or nursing staff considered analgesia necessary based on clinical assessment.

Perioperative complications included urinary leakage, urinary retention, and febrile UTI, and their severity was graded according to the Clavien–Dindo classification system. Double-J stents were placed postoperatively in patients who underwent ureteral tapering and in those who received bilateral ureteral reimplantation.

Urinary retention was defined as inability to void after catheter removal requiring recatheterization. Urine leakage was defined as persistent urinary drainage or imaging-confirmed urinary extravasation requiring prolonged drainage or catheterization. Recurrent UVJO was defined as recurrent or worsening hydroureteronephrosis with impaired drainage or declining renal function after surgery. Surgical success was defined as symptom resolution, improved or stable APD/UD, preserved DRF, and no recurrent UVJO or secondary intervention; otherwise, the outcome was considered surgical failure.

Follow-up outcomes included anteroposterior pelvic diameter (APD), ureteral diameter (UD), and DRF. New-onset VUR, recurrent UVJO, and secondary intervention during follow-up were also recorded as long-term complications. All patients underwent regular outpatient follow-up after surgery.

Ultrasonography was routinely performed at 1, 3, 6, and 12 months postoperatively and every 6–12 months thereafter. VCUG was performed 3–6 months after surgery to assess postoperative VUR. DMSA renal scintigraphy was generally performed 12 months after surgery to assess changes in DRF on the affected side.

APD was measured as the maximal anteroposterior diameter of the renal pelvis, and UD was measured as the maximal ureteral diameter on ultrasonography. Postoperative VUR was graded using the standard I–V grading system. Because of the retrospective design, imaging assessors were not formally blinded to surgical approach.

### 2.2. Statistical Analysis

Statistical analysis was performed using SPSS version 26.0 (IBM Corp., Armonk, NY, USA). Normality was assessed using the Shapiro–Wilk test. Continuous variables are presented as mean ± standard deviation or median (interquartile range), according to distribution, and categorical variables are presented as numbers and percentages. Between-group comparisons were performed using the independent-samples *t* test, Mann–Whitney U test, chi-square test, or Fisher’s exact test, as appropriate. Effect sizes with 95% confidence intervals (CI) were reported for major outcomes. Within-group preoperative-to-postoperative changes in APD, UD, and DRF were assessed using paired tests. No missing data were observed for the main outcomes; analyses were performed using complete cases. No formal adjustment for multiple comparisons was performed. Therefore, *p* values were interpreted descriptively and in conjunction with effect sizes and 95% CI. In bilateral cases, imaging and renal functional parameters from the more severely affected side were analyzed, whereas perioperative outcomes were analyzed per patient. Multivariable linear regression was used to evaluate operative duration after adjustment for age at surgery, preoperative UD, and year of surgery. A *p* value < 0.05 was considered statistically significant.

## 3. Results

### 3.1. Baseline Characteristics

**Figure 4:**
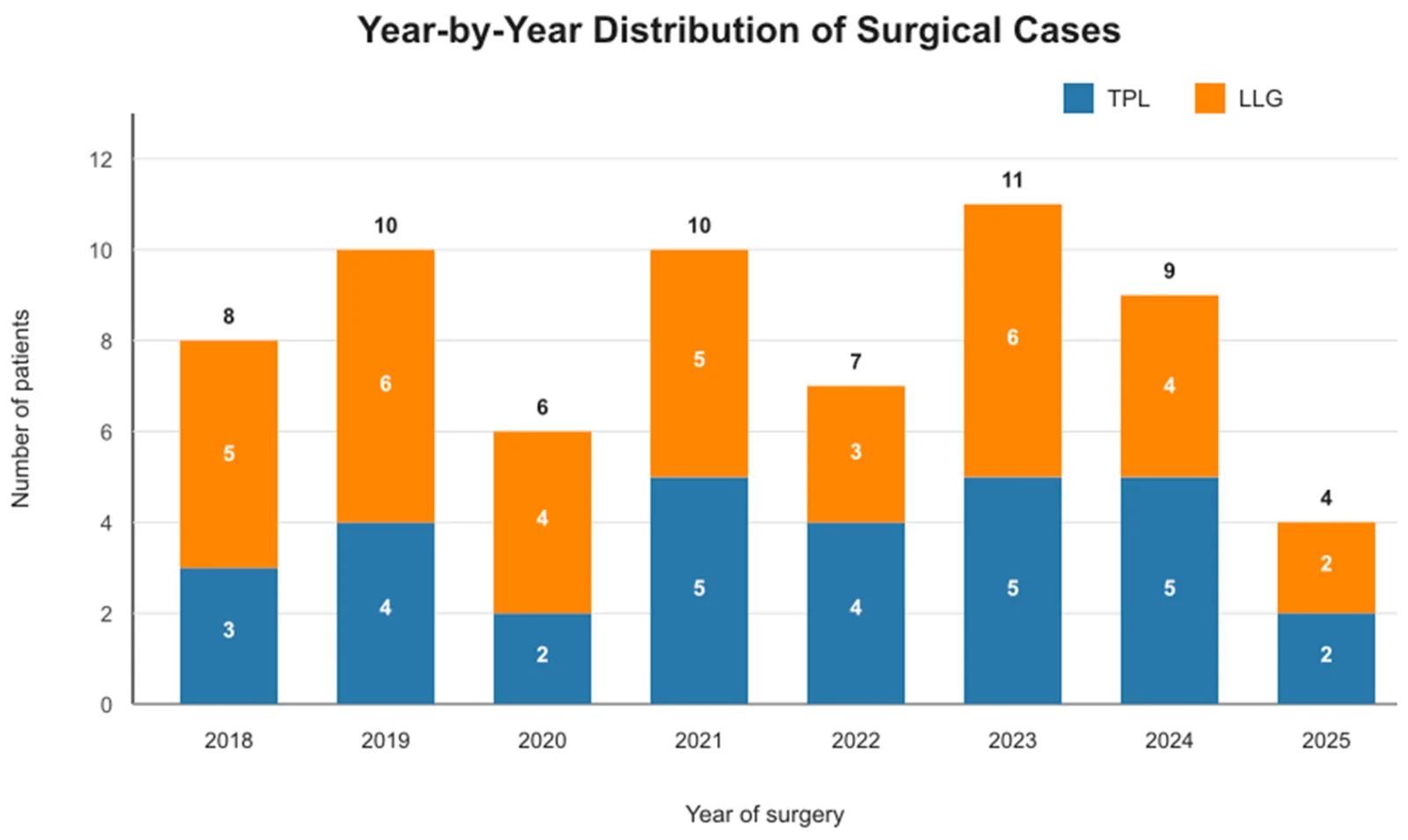
Year-by-year distribution of TPL and LLG cases from 2018 to 2025.

Baseline data are presented in Table 1. This study included 65 infants and toddlers with POM, comprising 30 in the TPL group and 35 in the LLG group. Prenatal detection was documented in 41 cases (63.1%), whereas 24 children (36.9%) were diagnosed after postnatal symptoms or clinical evaluation. In the TPL group, pre-reimplantation drainage included double-J stenting in 3 patients and percutaneous nephrostomy in 1 patient. In the LLG group, it included double-J stenting in 4 patients and percutaneous nephrostomy in 1 patient. No statistically significant differences were detected in age at surgery, body weight, sex distribution, laterality, diagnostic mode, or drainage before reimplantation. Baseline APD, UD, and DRF according to surgical indication are summarized in Supplementary Table S1. Cases were distributed from 2018 to 2025, and both TPL and LLG were performed throughout the study period (Figure 4).

### 3.2. Intraoperative Findings

**Figure 5:**
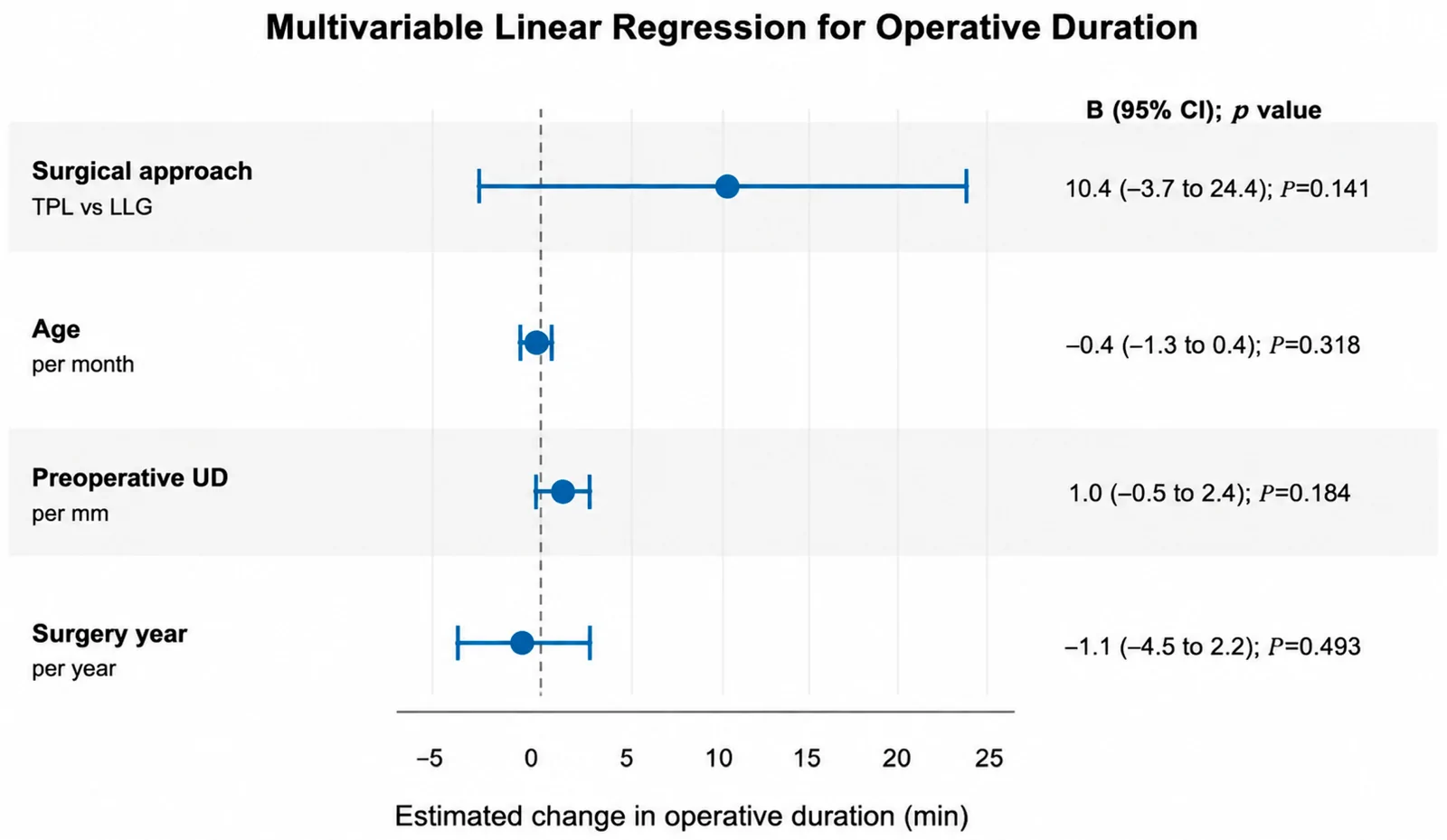
Forest plot of multivariable linear regression analysis for operative duration. Points represent regression coefficients (B), and horizontal lines indicate 95% CI. The dashed vertical line represents no estimated change (B = 0). Exact coefficients and *p* values are shown on the right.

All procedures were completed without open conversion. Total operative duration did not differ significantly between the TPL and LLG groups (145.8 ± 30.6 min vs. 136.9 ± 25.2 min; mean difference, 8.9 min; 95% CI, −4.9 to 22.7; *p* = 0.210; Table 2). In multivariable linear regression adjusted for age, UD, and surgery year, TPL showed a non-significant trend toward longer operative time than LLG (B = 10.4 min; 95% CI, −3.7 to 24.4; *p* = 0.141; Figure 5). No statistically significant differences were detected in estimated blood loss, ureteral tapering rate, or double-J stent use.

### 3.3. Perioperative Outcomes

Perioperative recovery and complications are shown in Table 2. Postoperative oral intake was resumed slightly earlier in the TPL group (6.1 ± 2.3 h vs. 8.2 ± 2.9 h; mean difference, −2.1 h; 95% CI, −3.4 to −0.8; *p* = 0.002). No statistically significant differences were detected in other postoperative recovery parameters, including FLACC pain score, acetaminophen use, duration of urinary catheterization, and postoperative hospital stay.

Perioperative complications were recorded in 8 patients, including 3 TPL patients and 5 LLG patients. In the TPL group, complications consisted of urinary leakage in one patient, which improved after prolonged catheterization and was classified as Clavien–Dindo grade I, and febrile UTI in two patients requiring antibiotic therapy, classified as grade II. In the LLG group, complications included urinary leakage in one patient managed with prolonged catheterization, urinary retention in two patients managed with temporary recatheterization, and febrile UTI in two patients requiring antibiotics. The urinary leakage and urinary retention cases were classified as grade I, whereas febrile UTI was classified as grade II.

### 3.4. Follow-Up Outcomes

Follow-up outcomes are summarized in Table 3. No statistically significant differences were detected between the two groups in follow-up duration, postoperative APD, postoperative UD, or postoperative DRF. In both groups, APD and UD decreased at the last follow-up, while DRF was preserved.

Low-grade postoperative VUR was detected in two unilateral left-sided LLG patients, including one grade I case at 4 months after surgery and one grade II case at 6 months after surgery. Both patients were managed conservatively with regular outpatient follow-up. No recurrent ureterovesical junction obstruction (UVJO), secondary intervention, or persistent symptoms despite radiographic improvement were observed in either unilateral or bilateral cases.

## 4. Discussion

The management strategy for POM in young children should be individualized according to clinical symptoms, the degree of upper urinary tract dilatation, and changes in DRF. Because some patients may experience spontaneous resolution or remain clinically stable during postnatal follow-up, initial conservative observation remains an important management approach. Previous British Association of Paediatric Urologists (BAPU) consensus recommendations also suggest that conservative management should be the initial treatment strategy for POM, while acknowledging the technical challenges associated with ureteral reimplantation during infancy [1]. In the present study, all patients had undergone outpatient follow-up and observation before surgery. Surgical intervention was performed because of recurrent febrile UTI, persistent or progressive hydroureteronephrosis, marked ureteral dilatation, or deterioration in DRF. Therefore, the study population more closely represents young children with POM who still required surgical correction after a period of conservative observation.

Our results showed marked reductions in APD and UD, while DRF was preserved during follow-up. These findings indicate postoperative improvement in upper urinary tract dilatation and preserved DRF in both groups during follow-up.

Transvesicoscopic ureteral reimplantation, as a minimally invasive approach in which ureteral reimplantation is completed intravesically, remains somewhat controversial in young children. Early experience from Children’s Hospital of Philadelphia suggested that younger age and smaller bladder capacity may increase the technical difficulty of transvesicoscopic surgery and the risk of complications [10]. However, with increasing surgical experience and technical refinements, subsequent studies have provided a more favorable assessment of transvesicoscopic ureteral reimplantation in young children. Benaired reported 60 children aged 6 months to 12 years who underwent transvesicoscopic Cohen ureteral reimplantation. All procedures were completed successfully, and the authors concluded that this technique could be applied in young children [11]. Similarly, Cui, in a study of infants with primary VUR, reported that all 31 procedures were completed successfully by experienced surgeons, with no conversion to open surgery. Although these studies primarily focused on VUR and the Cohen technique, their findings still suggest that the transvesicoscopic approach is safe and feasible for ureteral reimplantation in young children [12].

In recent years, TPL ureteral reimplantation has been increasingly applied for pediatric ureterovesical junction reconstruction, with favorable clinical outcomes reported [13,14,15]. Previous studies comparing transvesicoscopic Cohen and TPL procedures in children with VUR and POM have reported acceptable short-term outcomes for both techniques [16,17]. However, although the Cohen technique is well established and widely used, its cross-trigonal reimplantation route alters the natural course of the ureter and may make subsequent retrograde ureteral catheterization or ureteroscopic access more difficult. In contrast, the TPL technique reconstructs the ureterovesical junction by creating a longitudinal submucosal tunnel, thereby maintaining a ureteral orifice position and ureteral course that are relatively closer to physiological anatomy.

LLG ureteral reimplantation is an extravesical approach in which distal ureteral mobilization, management of the stenotic segment, ureteral tapering, and extravesical reimplantation are primarily performed within the abdominal cavity. This technique is less dependent on bladder capacity and provides a relatively wider operative field in the abdominal cavity. Therefore, it has been more commonly adopted in the minimally invasive treatment of POM in young children and has achieved favorable outcomes [18,19]. However, the LLG technique requires extravesical ureteral dissection and detrusor tunnel incision. In cases requiring bilateral procedures or extensive ureteral mobilization, a potential risk of injury to the detrusor neurovascular supply remains, which may lead to subsequent postoperative voiding dysfunction [20,21]. In addition, for markedly dilated and tortuous megaureters, performing ureteral tapering, reconstruction, and reimplantation within the limited pelvic space also requires advanced intracorporeal suturing ability and anatomical orientation.

Maintaining a stable pneumovesical working space is critical for the successful completion of TPL ureteral reimplantation in young children. First, proper trocar placement and secure fixation are essential. Because young children have a small bladder capacity and thin bladder wall, trocar displacement or peritrocar gas leakage can rapidly lead to loss of the pneumovesical space and an unstable operative field. Therefore, trocars should be inserted under direct vision after adequate bladder distension. Bladder wall suspension and abdominal wall fixation sutures should also be used to enhance port stability and reduce the risk of trocar dislodgement and gas leakage.

Second, during distal ureteral mobilization, CO_2_ may enter the extravesical or peritoneal space through local tissue planes, further compromising the pneumovesical working space. To minimize this problem, meticulous dissection should be performed as close as possible to the ureteral surface, while avoiding excessive separation of extravesical tissue planes. If gas escape occurs, extraperitoneal or intraperitoneal gas can be promptly decompressed by inserting a syringe needle through the umbilicus or abdominal wall, and the intravesical pressure can be adjusted as needed to restore a stable operative field. In the present study, transient gas escape occurred in three patients in the TPL group. After the above measures were applied, a stable working space was restored in all cases.

With regard to operative time, previous comparative studies on POM have suggested that the transvesicoscopic approach may be associated with a longer operative duration [8,9,22]. However, a prolonged operative time may not be inherent to the transvesicoscopic approach. Previous studies of TPL ureteral reimplantation have suggested that operative time may decrease with increasing surgical experience [23]. In our cohort, no statistically significant difference in operative time was detected between the two groups. In the adjusted analysis, TPL showed a non-significant trend toward longer operative time.

In the present study, the TPL group resumed oral intake slightly earlier than the LLG group. This may be partly related to the intravesical nature of the TPL approach, which avoids entry into the peritoneal cavity and may reduce peritoneal and bowel irritation. However, postoperative feeding was not managed according to a standardized protocol; therefore, the clinical relevance of this difference remains uncertain and should be interpreted cautiously. In addition, no statistically significant differences were detected in FLACC pain scores, analgesic use, duration of urinary catheterization, or postoperative length of stay. Taken together, these findings do not demonstrate a clear difference in overall early postoperative recovery between the two approaches.

No statistically significant difference in postoperative complication rates was detected between the two groups, and no severe complications requiring reoperation occurred in either group. Observed complications, including postoperative UTI, urinary retention, urinary leakage, and new-onset low-grade VUR, were manageable with conservative treatment or antibiotic therapy. Previous comparative studies on POM have also shown no obvious difference in the overall complication rate between the transvesicoscopic approach and the laparoscopic extravesical approach, and our results showed a similar pattern [8,9].

Several limitations should be acknowledged. First, this was a single-center retrospective study with nonrandomized and unadjusted treatment allocation, and residual confounding related to surgeon preference, anatomical complexity, bilateral disease, preoperative drainage, and surgical indication could not be fully excluded. Second, the sample size was small, and the follow-up duration was limited. Third, the learning curve, surgeon-specific expertise, and possible temporal bias may have influenced the results. Fourth, imaging assessments were not formally blinded to the surgical approach. Fifth, no formal power calculation was performed, and this study was not designed within an equivalence or noninferiority framework. Therefore, the absence of statistically significant differences should not be interpreted as evidence of equivalence. Finally, the limited sample size and follow-up duration reduced the ability to detect rare or late complications.

## 5. Conclusions

In this single-center retrospective cohort, no statistically significant differences were detected between TPL and LLG in operative time, perioperative complications, or follow-up outcomes. TPL ureteral reimplantation allows reconstruction to be completed intravesically while maintaining a ureteral course that more closely approximates the natural anatomy, and it may represent an alternative minimally invasive option for selected infants and toddlers with POM. Larger prospective studies with longer follow-up are needed to further evaluate its long-term safety and efficacy.

## Figures and Tables

**Table 1:** Baseline demographic and clinical profiles of the TPL and LLG groups.

Variable	TPL Group (*n* = 30)	LLG Group (*n* = 35)	Effect Size (95% CI)	*p* Value
Age (months)	19.8 ± 9.3	16.6 ± 7.9	3.2 (−1.0 to 7.4)	0.148
Weight (kg)	11.3 ± 2.2	10.5 ± 1.9	0.8 (−0.2 to 1.8)	0.156
Gender, *n* (%)			—	0.745
Male	23 (76.7%)	28 (80.0%)		
Female	7 (23.3%)	7 (20.0%)		
Laterality, *n* (%)			—	0.725
Left	21 (70.0%)	22 (62.9%)		
Right	6 (20.0%)	10 (28.6%)		
Bilateral	3 (10.0%)	3 (8.6%)		
Mode of diagnosis, *n* (%)			—	0.579
Prenatal diagnosis	20 (66.7%)	21 (60.0%)		
Postnatal diagnosis	10 (33.3%)	14 (40.0%)		
Surgical indication, *n* (%)			—	0.913
Progressive hydroureteronephrosis	16 (53.3%)	18 (51.4%)		
Recurrent febrile UTI	10 (33.3%)	11 (31.4%)		
DRF impairment/decline	4 (13.3%)	6 (17.1%)		
Pre-reimplantation drainage, *n* (%)	4 (13.3%)	5 (14.3%)	—	0.912

Values are presented as mean ± standard deviation or *n* (%). Percentages were calculated per patient.

**Table 2:** Perioperative outcomes and complications in the TPL and LLG groups.

Variable	TPL Group (*n* = 30)	LLG Group (*n* = 35)	Effect Size (95% CI)	*p* Value
Total operative duration (min)	145.8 ± 30.6	136.9 ± 25.2	8.9 (−4.9 to 22.7)	0.210
Estimated blood loss (mL)	8.2 ± 3.6	8.7 ± 4.2	−0.5 (−2.4 to 1.4)	0.612
Ureteral tapering, *n* (%)	15 (50.0%)	17 (48.6%)	—	0.909
Double-J stent placement, *n* (%)	16 (53.3%)	18 (51.4%)	—	0.878
Time to oral intake (h)	6.1 ± 2.3	8.2 ± 2.9	−2.1 (−3.4 to −0.8)	0.002
FLACC pain score on day 1	2.0 ± 0.7	2.1 ± 0.8	−0.1 (−0.5 to 0.3)	0.636
Number of acetaminophen doses	1.0 (1.0, 2.0)	1.0 (1.0, 2.0)	0.0 (−1.0 to 1.0)	0.567
Urinary catheterization duration (days)	3.0 (2.0, 4.0)	3.0 (2.0, 3.0)	0.0 (0.0 to 0.5)	0.421
Postoperative hospital stay (days)	4.0 (3.0, 5.0)	4.0 (4.0, 5.0)	0.0 (−1.0 to 0.5)	0.634
Perioperative complications, *n* (%)	3 (10.0%)	5 (14.3%)	−4.3 (−20.1% to 11.5%)	0.716
Urine leakage (Grade I)	1 (3.3%)	1 (2.9%)		
Urinary retention (Grade I)	0	2 (5.7%)		
UTI (Grade II)	2 (6.7%)	2 (5.7%)		

Values are presented as mean ± standard deviation, median (interquartile range), or *n* (%). All perioperative outcomes and complications were analyzed per patient.

**Table 3:** Follow-up outcomes and long-term complications in the TPL and LLG groups.

Variable	TPL Group (*n* = 30)	LLG Group (*n* = 35)	Effect Size (95% CI)	*p* Value
Follow-up duration (months)	40.0 ± 17.4	46.5 ± 21.1	−6.5 (−15.9 to 2.9)	0.179
Preoperative APD (mm)	22.1 ± 7.1	21.2 ± 6.6	0.9 (−2.5 to 4.3)	0.610
Postoperative APD (mm)	9.0 ± 3.1	10.0 ± 3.9	−1.0 (−2.7 to 0.7)	0.261
Change in APD (mm)	−13.1 ± 6.2	−11.2 ± 5.7	−1.9 (−4.9 to 1.1)	0.216
Preoperative UD (mm)	15.1 ± 5.2	14.6 ± 4.7	0.5 (−2.0 to 2.9)	0.654
Postoperative UD (mm)	6.2 ± 2.7	5.9 ± 2.4	0.3 (−1.0 to 1.6)	0.649
Change in UD (mm)	−8.9 ± 2.7	−8.7 ± 2.5	−0.2 (−1.5 to 1.1)	0.743
Preoperative DRF (%)	38.0 ± 4.9	38.8 ± 4.2	−0.8 (−3.0 to 1.4)	0.459
Postoperative DRF (%)	40.3 ± 4.3	40.9 ± 4.4	−0.6 (−2.7 to 1.5)	0.607
Change in DRF (%)	2.3 ± 1.2	2.1 ± 1.1	−0.2 (−1.5 to 1.1)	0.394
Long-term complications, *n* (%)	0	2 (5.7%)	−5.7% (−13.4% to 2.0%)	0.496
VUR	0	2 (5.7%)		
UVJO	0	0		
Secondary intervention	0	0		

Values are presented as mean ± standard deviation or *n* (%). Imaging and renal functional parameters were analyzed on the affected side in unilateral cases and on the more severely affected side in bilateral cases. Long-term complications were analyzed per patient.

## Data Availability

The data and materials can be obtained by contacting the corresponding author.

## Supplementary Materials

The following supporting information can be downloaded at: https://www.mdpi.com/article/10.3390/jcm15166178/s1, Table S1: Baseline APD, UD, and DRF according to surgical indication.
