# Supplementary material for: Transvesicoscopic Politano–Leadbetter Versus Laparoscopic Lich–Gregoir Ureteral Reimplantation for Primary Obstructive Megaureter in Infants and Toddlers: A Retrospective Comparative Cohort Study
Source: J Clin Med. 2026 Aug 10;15(16):6178. doi: 10.3390/jcm15166178 (PMC13513172; doi:10.3390/jcm15166178)
Supplement: Supplementary file 1 [file jcm-15-06178-s001.zip › jcm-4383239-supplementary.pdf]

Supplementary Table S1. Baseline APD, UD, and DRF according to surgical indication

Surgical indication	Group	n	APD,mm	UD, mm	DRF, %
Progressive hydroureteronephrosis	TPL	16	23.6 ± 7.8	15.5 ± 5.9	38.1 ± 4.5
	LLG	18	22.7 ± 6.7	15.2 ± 5.5	38.9 ± 4.4
Recurrent febrile UTI	TPL	10	20.5 ± 6.7	14.7 ± 3.7	38.5 ± 3.0
	LLG	11	19.8 ± 6.4	14.3 ± 2.7	39.5 ± 2.2
DRF impairment/decline	TPL	4	20.0 ± 4.5	14.5 ± 6.6	36.0 ± 8.1
	LLG	6	19.5 ± 6.9	13.5 ± 5.8	37.0 ± 6.3
